# Functional Elucidation of *Vitellogenin receptor* Activity in *Apis mellifera* in Response to Abiotic Stress

**DOI:** 10.3390/insects16070650

**Published:** 2025-06-21

**Authors:** Li Lei, Hongyu Song, Zhenguo Liu, Ge Zhang, Ying Wang, Baohua Xu

**Affiliations:** College of Animal Science and Technology, Shandong Agricultural University, Tai’an 271018, China; leili_halcyon@163.com (L.L.); songhy2@163.com (H.S.); zgliu@sdau.edu.cn (Z.L.); gezhang.bee@gmail.com (G.Z.); wangying@sdau.edu.cn (Y.W.)

**Keywords:** *Apis mellifera*, *AmVgR*, oxidative stress, heavy metal, pesticide stress

## Abstract

Abiotic stressors threaten honeybee health, jeopardizing pollination services critical to agriculture and biodiversity. Here, we identified the *AmVgR* gene, which encodes a member of the low-density lipoprotein receptor family, and examined its function in the response of *Apis mellifera* to adverse abiotic stress. *AmVgR* exhibited peak expression in adult workers and was significantly upregulated under heat, cold, heavy metal, and pesticide exposure. RNAi-mediated knockdown of *AmVgR* suppressed antioxidant enzyme activities, elevated the levels of oxidative damage markers, and downregulated antioxidant gene expression. Crucially, *AmVgR* silencing reduced survival under H_2_O_2_-induced oxidative stress, indicating its essential role in stress resilience. Our findings highlight *AmVgR* as a key regulator of antioxidant defense during development and environmental adaptation in *Apis mellifera*. This study provides mechanistic insights into bee stress physiology and proposes *AmVgR* as a novel target for enhancing pollinator protection strategies. Further research should elucidate its molecular pathways and translational applications in mitigating abiotic stress impacts.

## Simple Summary

Environmental stressors like extreme temperatures, pesticides, and heavy metals threaten honeybee health. This study identifies *AmVgR* as critical for protecting *Apis mellifera* from oxidative stress. *AmVgR* is highly expressed in adult workers and further upregulated under stress. Silencing *AmVgR* via RNAi reduced the antioxidant activity, increased oxidative damage, and lowered survival under oxidative stress. These findings demonstrate *AmVgR* essential role in bee stress resilience, offering a novel target to enhance pollinator conservation strategies.

## 1. Introduction

Bees are primary pollinators in ecosystems, playing a crucial role in agricultural productivity and ecological balance [1]. However, over the past few decades, bee populations have sharply declined, negatively impacting ecosystem stability, crop yields, and societal benefits [2,3]. Growing evidence suggests that various environmental stressors are key factors contributing to the sharp decline in bee populations [4,5,6]. Therefore, investigating the adverse effects of oxidative stressors, such as temperature and pesticides, on bees has become a focal point in environmental studies. For example, when bees go out for foraging, they are often stimulated by abiotic stress factors such as high and low temperatures, UV radiation, heavy metals, and pesticides [7], which triggers oxidative stress. The occurrence of oxidative stress can lead to damage to nucleic acids and proteins in organisms, and in severe cases, it can endanger life [8]. High temperatures cause a continuous increase in metabolic rate and oxygen demand in bees, thereby inducing the production of reactive oxygen species (ROS) and toxic substances, which causes cell damage and affects the health of bees [9]. Pesticides such as imidacloprid and thiamethoxam can participate in the redox cycle after entering the body of bees, causing oxidative stress and resulting in an imbalance in ROS metabolism. They can also reduce the antioxidant capacity of insects such as bees by decreasing the activity of antioxidants and related enzymes or consuming some reducing agents in cells, which seriously affects the survival of bees [10,11]. Therefore, the adverse effects of oxidative stressors such as extreme temperatures and insecticides on bees have become the focus of environmental research.

Bees trigger their own defense responses in adverse environments. Through their inherent antioxidant mechanisms, they produce antioxidants to alleviate oxidative damage caused by stress [7]. For example, metabolic enzymes such as cytochrome P450 (CYP450) and glutathione S-transferase (GST) directly decompose pesticides and their secondary metabolites [12,13]. In addition, to avoid the adverse effects of ROS caused by pesticides, bees can also utilize antioxidant proteins such as superoxide dismutase (SOD) and catalase (CAT) to reduce the production of ROS, thereby mitigating the oxidative stress caused by pesticides [14]. Therefore, exploring antioxidant stress-related proteins in bees is of great significance for enhancing their antioxidant capacity and resistance to external stress factors.

In Park’s study, *Vitellogenin* (*Vg*) was shown to protect DNA from ROS damage and participate in regulating the defense system against ROS, functioning as an antioxidant within the organism [15]. In *Aedes aegypti* and *Danaus plexippus*, the level of *Vg* increased significantly when these bees were exposed to extreme temperatures and heavy metal-polluted environments [16,17,18]. In *alfalfa leafcutting*, the expression of *Vg* was positively correlated with the activity of antioxidant enzymes, suggesting that *Vg* may protect cells from oxidative damage by enhancing the expression of antioxidant enzymes [19]. In honeybees, the expression of *Vg* was closely associated with the gene expression of antioxidant enzymes, which could effectively reduce oxidative stress parameters, thereby enhancing the hygienic and cleaning behaviors of honeybees [20]. The functions of *Vg* in insects are not limited to the synthesis of yolk proteins but are also closely related to antioxidant stress responses, especially those involving the glutathione system [21].

The *Vitellogenin receptor* (*VgR*) is the specific receptor for the endocytosis of *Vg*. Its main function is to transport *Vg* into the oocytes. After a series of modifications and processing, it forms and accumulates vitellin in the oocytes, providing sufficient nutrients and the required energy for the embryonic development of insects [22,23,24,25,26]. *VgR* belongs to the low-density lipoprotein receptor (LDLR) family. Its molecular structure usually consists of multiple domains, including the ligand-binding domain (LBD), EGF-precursor homology domain (EGF-PHD), O-linked carbohydrate domain (OLCD), transmembrane domain (TD), and cytoplasmic domain (CyD) [23,27]. The combination of these domains enables *VgR* to effectively bind to its ligand Vg and transmit signals [28]. Research has shown that *VgR* is not only detectable in insect ovaries but also expressed in various tissues of many insects, especially the hypopharyngeal glands, fat body, and midgut of bees [29], indicating that the ovary may not be the only organ where *VgR* exerts its functions. Reproductive performance in insects often interplays with immune performance, a relationship regulated by resource allocation, environmental stress, and pathogen exposure [30]. For example, in Orthoptera, induction of the immune system with heat-killed bacteria or bacterial cell wall components reduces egg production in *Gryllus texensis* [31], *Hemideina crassidens* [32], and *Acheta domesticus* [33]. Similarly, in the genus Anopheles, exposure to bacterial cell wall components or infection by *Plasmodium* spp. results in significant reductions in protein accumulation within the ovaries, promotes apoptosis of follicle cells, and decreases the number of eggs laid [34]. Therefore, we hypothesized that *VgR* may not only regulate insect reproductive performance but also play a significant role in protecting bees from oxidative stress resulting from abiotic environmental stress.

This study isolated and identified the *AmVgR* gene from *Apis mellifera*, investigating its role in oxidative stress induced by abiotic stressors in *Apis mellifera*. The structural and functional predictions of the encoded protein were analyzed, comprehensively exploring the role of *AmVgR* in the antioxidant stress response of honeybees to abiotic stressors such as temperature, pesticides, and heavy metals at physiological, biochemical, and molecular levels. We anticipate that the results of this study will provide new insights into the function of *AmVgR* and offer theoretical knowledge for elucidating the role of *AmVgR* in the organism’s resistance to abiotic stress.

## 2. Materials and Methods

### 2.1. Insect Sample Collection and Processing

*Apis mellifera* specimens were sourced from the College of Animal Science and Technology at Shandong Agricultural University in Tai’an, China. We selected 2–3 healthy bee colonies, marked 1-day-old eggs, and put them back into the colonies for hatching and rearing; they were used for the subsequent collection of bee samples at different ages. We randomly collected larvae aged 4–6 days (L4–L6), white-eyed pupae (Pw), brown-eyed pupae (Pb), and black-eyed pupae (Pd). We marked the thoraxes of newly emerged worker bees with paint, randomly caught worker bees aged 1–21 days (A1–A21), placed 16-day-old worker bees (A16) on an ice box, dissected them, and collected tissue samples from the head, thorax, and abdomen. We randomly caught 450 forager bees and placed them in wooden beehives (150 × 150 × 50 mm), then randomly divided them into 9 groups of 50 bees each. Three groups were subjected to high-temperature (45 °C), low-temperature (4 °C), and UV treatments by placing the bees in high- and low-temperature, constant-temperature incubators and under ultraviolet light irradiation, and samples were taken after 0, 1, 2, 3, 4, and 5 h for subsequent experiments. Three groups were subjected to oxidative stress treatments with H_2_O_2_ (2 mol/L), CdCl_2_ (1.7 μL/mL), and HgCl_2_ (1.7 μL/mL), which involved feeding the bees with oxidants diluted to the corresponding concentrations with a 50% (*w*/*v*) sucrose solution. The other three groups were exposed to pesticide stress treatments with imidacloprid (0.02 mg/mL), thiamethoxam (0.03 mg/mL), and haloxyfop-R-methyl (6 μL/mL) by feeding each group of bees with a 50% (*w*/*v*) sucrose solution containing the corresponding pesticide concentrations, and samples were also taken at 0, 1, 2, 3, 4, and 5 h for subsequent experiments. For the bees in the oxidant and pesticide treatment groups, the sucrose solutions containing the corresponding concentrations of oxidants and pesticides were placed in 1/2 of a 96-well sterile cell culture plate and put in the wooden beehives for the bees to feed freely. The complete chemical names, purities, sources, and treatment concentrations of all pesticides and heavy metals used by IUPAC are shown in Table S1. The bees were reared in a breeding box with a relative humidity of 70% and a temperature of 33 °C, which is consistent with the natural bee colony rearing method and the behavioral patterns of bees.

### 2.2. RNA Extraction, cDNA Synthesis, and AmVgR Gene Cloning

Total RNA was extracted from the collected *Apis mellifera* samples using the TransZol Up Plus RNA Kit (ER501-01-V2, TransGen, Beijing, China). After the concentration and purity of the total RNA were determined by a spectrophotometer, cDNA was synthesized using the EasyScript^®^ One-Step gDNA Removal and cDNA Synthesis SuperMix (AE311-02, TransGen, Beijing, China). Based on the transcription data of the *AmVgR* gene provided by the National Center for Biotechnology Information (GenBank: XM_026439867.1), primers were designed in the functional region of the *AmVgR* gene using Primer5.0 software. The primer sequences are presented in Table S2 and were synthesized by Sangon Biotech (Shanghai) Co., Ltd. (Shanghai, China). The specific fragment of *AmVgR* was amplified with the cDNA of *Apis mellifera* as the template. The amplification system included 2 μL of forward primer, 2 μL of reverse primer, 25 μL of 2× Rapid Taq MasterMix, 2 μL of cDNA, and 19 μL of ddH_2_O, in a total volume of 50 μL. The detailed amplification protocol is shown in Table S3. The reaction products were separated on a 2% agarose gel. Subsequently, the target gene was purified by the SteadyPure Agarose Gel DNA Purification Kit (AG21005, Biotechnology (Hunan) Co., Ltd., Changsha, China).

### 2.3. Real-Time Quantitative Polymerase Chain Reaction Analysis (RT-qPCR)

The RT-qPCR analysis was performed using the CFX96 Real-Time Detection System and SYBR Green Premix Pro Tag HS qPCR Kit (AG11718, Biotechnology (Hunan) Co., Ltd., Changsha, China). The reference gene was the stably expressed β-actin gene (GenBank accession number: NM_001185146). The reaction system included SYBR^®^ Green Premix Pro Taq HS Premix 10 μL, DNA template 2 μL, Primer F (10 μM) 0.4 μL, Primer R (10 μM) 0.4 μL, and RNase free water 7.2 μL, in a total volume of 20 μL. The reaction protocol is shown in Table S4. The primer sequences are shown in Table S2, and the primers were synthesized by Shanghai Sangon Biotech Co., Ltd.

### 2.4. Anti-AmVgR Antibody Preparation

Construction of the pET-30a (+)-*AmVgR* plasmid [35]. Briefly, the CDS region of *AmVgR* was amplified using primers containing the BamH I and Sal I restriction enzyme sites. After digestion with the same restriction enzymes, it was inserted into the expression vector pET-30a (+) (Novagen, Darmstadt, Germany) at the same enzyme-cut sites. Then, the pET-30a (+) expression plasmid was introduced into BL21 (DE3) cells. Protein expression was induced with 0.2 mM isopropyl-1-thio-ß-D-galactopyranoside (IPTG) at 28 °C. After 8–10 h, the cells entered the logarithmic growth phase. The recombinant pET-30a (+) protein was purified using the method described by Zhang et al. (2013) [36] and detected by 12% sodium dodecyl sulfate-polyacrylamide gel electrophoresis (SDS-PAGE) [36]. After detection, the purified protein was subcutaneously injected into healthy female New Zealand white rabbits (4-month-old) to generate antibodies. After multiple immunizations, blood was collected from the carotid artery. Serum was extracted, and the antibody titer was determined by ELISA. Then, the anti-AmVgR serum was stored at −80 °C for subsequent Western blot analyses. The antibody preparation and testing steps were all completed by Sangon Biotech (Shanghai) Co., Ltd. (Shanghai, China).

### 2.5. Western Blot Analyses (WB)

Based on the amino acid sequence of the vitellogenin receptor of *Apis mellifera*, comprehensive analyses of its signal peptide (https://services.healthtech.dtu.dk/services/SignalP-5.0/, accessed on 26 July 2021), transmembrane domain (http://www.cbs.dtu.dk/services/TMHMM-2.0/, accessed on 26 July 2021), and hydrophilicity (https://web.expasy.org/protscale/, accessed on 26 July 2021) were conducted using software. A polypeptide fragment with the sequence DCPDKSDEEKCEN was designed. Sangon Biotech (Shanghai) Co., Ltd. (Shanghai, China), was commissioned to prepare rabbit polyclonal antibodies against the AmVgR protein. WB was used to detect changes in the AmVgR protein level under different stress treatments. Total protein lysates were prepared from A16 worker bees exposed to various stresses (4 °C, 45 °C, and UV) using a tissue protein extraction kit (CWbiotech, Beijing, China). Then, the obtained total protein lysates were subjected to SDS-PAGE at a loading amount of 50 μg per well. Subsequently, the target bands were excised and electro-transferred onto a PVDF membrane (Millipore, Bedford, MA, USA) in a semi-dry transfer device. Three replicate blots were generated for each sample. Anti-AmVgR serum diluted at 1:1000 (*v*/*v*) was used as the primary antibody. Peroxidase-labeled goat anti-mouse immunoglobulin G (Dingguo, Beijing, China) was used as the secondary antibody, diluted 1:2000 (*v*/*v*), and mouse anti-α-tubulin (1:2000) was also used as a secondary antibody. α-Tubulin was used for normalization purposes. The development process was carried out using the FDbio-Dura ECL kit (Fudebio, Hangzhou, China). The results of the Western blot were analyzed using Image-Pro Plus 6.0 (Media Cybernetics, Rockville, MD, USA).

### 2.6. Bioinformatics Analysis of the AmVgR Gene

The open reading frame (ORF) of the *AmVgR* gene was analyzed, and its amino acid sequence was translated using the ORF Finder software. The relative molecular mass (MW) and theoretical isoelectric point (pI) were evaluated using the ExPasy-ProtParam tool (https://web.expasy.org/protparam/, accessed on 1 July 2021). The tertiary structure of the *AmVgR* protein was predicted via the SWISS-MODEL online website (https://swissmodel.expasy.org/, accessed on 1 September 2022), and the protein sequence map of *AmVgR* was drawn using the IBS-1.0.3 software. The binding domains and catalytic residues were predicted through Conserved Domain Search (https://www.ncbi.nlm.nih.gov/Structure/cdd/cdd.shtml, accessed on 1 September 2022). Subsequently, the amino acid sequences of homologous *VgR*s were downloaded from GenBank. A phylogenetic tree was constructed using the neighbor-joining method with 1000 bootstrap replicates in MEGA 7-X software (Table S5).

### 2.7. RNA Interference of the AmVgR Gene

To further explore the *AmVgR* gene and verify its function, we amplified a silencing fragment using primers corresponding to the T7 promoter sequence (Table S2). The gene silencing fragment was purified by gel extraction using an agarose gel DNA recovery kit (Magen, China) and used as the double-stranded template for synthesizing the control group *dsGFP* and the experimental group *dsAmVgR*. Then, dsRNA was prepared according to the instructions of the T7 high-efficiency transcription kit (TR102, Vazyme Biotech Co., Ltd., Nanjing, China). *GFP* was used as a control (GenBank accession number: U87974). We caught A16 forager bees. The bees were taken out of the beehive, gently held by the wings with hands, and fed with 2.5 μL of *dsRNA* (2 μg/μL) using a pipette. Fifty bees were fed with *dsGFP* in the control group, and another fifty with *dsAmVgR* in the treatment group. Samples were collected after 48 h for the determination of gene silencing efficiency.

### 2.8. Expression Profiling of Antioxidant Genes After RNAi Treatment

RT-qPCR was used to detect the transcriptional levels of the antioxidant-related genes *GTPX*, *GSTO1*, *Tpx3*, *Tpx5*, *CAT*, *SOD1*, *CYP450*, and *MSRA* after gene silencing. *β-Actin* was used as an internal reference gene for the quantitative analysis, and the primers used are shown in Table S2.

### 2.9. Determination of Antioxidant Enzyme Activity and Metabolites After RNAi Treatment

The bee samples after gene silencing were placed in a certain volume of PBS at a mass ratio of 1:9 for tissue homogenization. The resulting homogenate was subjected to centrifugation at 2500 rpm at 4 °C for 20 min, after which the supernatant was collected. Enzyme-linked immunosorbent assay kits (MLBIO Co., Shanghai, China) were employed to quantify catalase (CAT), superoxide dismutase (SOD), and total antioxidant capacity (T-AOC) in the supernatant, adhering strictly to the manufacturer’s protocols. The final results were normalized against the protein concentration of each sample. Additionally, the levels of vitamin C (VC, A009-1-1), malondialdehyde (MDA, A003-1-2), and hydrogen peroxide (H_2_O_2_, A064-1-1) in the supernatant were assessed using kits obtained from Nanjing Jiancheng Bioengineering Institute.

### 2.10. Detection of Oxidative Stress Tolerance in Bees After RNAi Treatment

To investigate the effect of the *AmVgR* gene on oxidative stress in bees, we fed the bees with a 50% sucrose solution containing H_2_O_2_ (2 mol/L) 48 h after interference of the *AmVgR* gene. The number of dead bees was recorded at 2, 6, and 12 h after H_2_O_2_ treatment, with 50 bees in each group. All bees were maintained in a dark environment at 33 °C and 70% humidity.

### 2.11. Data Analysis

Data analysis was performed using SPSS statistical software (IBM SPSS Statistics 25, Chicago, IL, USA). Tukey’s honestly significant difference test (Tukey’s HSD) was used for pairwise comparisons between groups to determine significant differences, while one-way analysis of variance (ANOVA) was employed for comparisons among multiple groups. The data are presented as the mean ± SEM (n = 3). In the figures, different letters indicate significant differences (*p* < 0.05), and the same letters indicate non-significant differences (*p* > 0.05).

## 3. Results

### 3.1. AmVgR Gene Isolation and Structure Specificity Analysis

**Figure 1:**
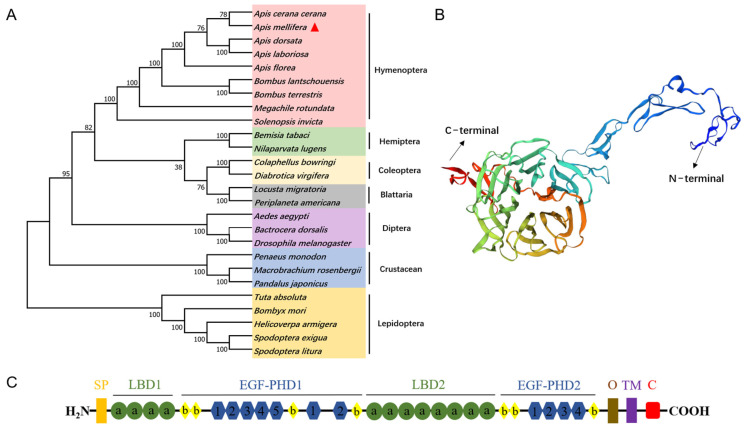
*AmVgR* bioinformatics analysis. (**A**) Phylogenetic tree of *VgR* in *Apis mellifera* and other insects. (**B**) Tertiary structure of *AmVgR* proteins predicted by SWISS-M*ODEL*. (**C**) Conservative domain analysis of *AmVgR*. SP: signal peptide; LBD1-2: ligand-binding domain; EGF-PHD1-2: EGF-precursor homology domain; a: LDLR class A repeat (LDLRa); b: LDLR class B repeat (LDLRb); 1–5: YWXD motif; O: potential O-linked sugar domain; TM: transmembrane domain; C: cytoplasmic domain. *AmVgR* is marked by a red triangle, and all *VgR* amino acid sequences were downloaded from GenBank (the accession numbers and species names are listed in Table S5).

The full-length cDNA sequence of *AmVgR* (Gene symbol: LOC725920) was obtained by RT-qPCR. The *AmVgR* cloning results showed that the *AmVgR* amplified bands were consistent in size with the predicted 5265 bp target gene, and the base sequences of the encoded genes were identical by sequence comparison, indicating that the *AmVgR* gene was successfully cloned (Figure S1). To investigate the similarity of *AmVgR* in different species, we conducted an evolutionary tree construction by MEGA-X using the proximity method to analyze the homology of different genera of insects in Hymenoptera. The results showed that *AmVgR* had high homology in different insects, with the highest homology in *Apis cerana cerana,* followed by hemipteran insects (Figure 1A). To explore the structure of *AmVgR*, we predicted its protein structure by the online protein structure domain prediction tools SwissModel and Swiss-PdbViewer 4.1.0. The results showed that *AmVgR* encodes a protein containing 1754 amino acids (Figure 1B). The predicted pI and MW of the deduced protein appeared to be 5.38 and 198.3 kDa, respectively, using the online tool ProtParam. Domain analysis revealed that *AmVgR* from *Apis mellifera* has two LBDs, with four LDLRa (class A) cysteine-rich repeats in the first ligand-binding domain (LBD) and eight repeats in the second LBD (Figure 1C). SignalP-5.0 was used to predict the signal peptide sequence of *AmVgR* from *Apis mellifera*, revealing that *AmVgR* encodes a signal peptide consisting of 18 amino acids at the N-terminus: MSRNLLVFFVLTNFYCSS (Figure S2).

### 3.2. Identification and Analysis of the Promoter Region

**Figure 2:**
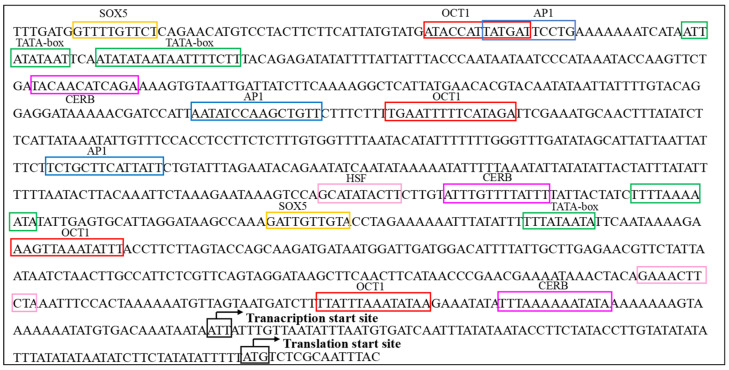
Prediction and analysis of cis-acting elements in the promoter sequence of the *AmVgR* gene. The transcription and translation start sites are indicated by black arrows, and different cis-acting elements related to oxidative stress and development are marked with different colors. Yellow box: SRY-box transcription factor 5 (SOX5); red box: octamer-binding transcription factor 1 (OCT1); blue box: activating protein-1 (AP1); green box: TATA box; pink box: heat-shock factors (HSFs); purple box: cAMP response element binding protein (CREB).

To deepen our understanding of the structural functionality of *AmVgR*, we performed a comprehensive analysis of its promoter region. We utilized the TFBIND online tool (http://tfbind.hgc.jp/, accessed on 1 September 2022) to scrutinize a 1000 bp promoter sequence acquired from NCBI, aiming to clarify the transcriptional regulation mechanisms associated with *AmVgR*. This examination uncovered a variety of transcription factor binding sites (TFBSs) (Figure 2). Furthermore, we extended our analysis by employing the same TFBIND software to evaluate an additional 1123 bp promoter sequence sourced from NCBI, which led to the identification of several more TFBSs (Figure 2). Notably, a number of transcription factors that are crucial in mediating responses to environmental stress and immune functions were recognized, including activating protein-1 (AP1) [37], heat-shock factors (HSFs) [38,39], and cAMP response element binding protein (CREB) [40]. Moreover, several transcription factors pertinent to tissue development were identified within the promoter region of *AmVgR*, specifically, caudal-related homeobox, SRY-box transcription factor 5 (Sox-5) [41], and octamer-binding transcription factor 1 (Oct-1) [42].

### 3.3. Analysis of the Spatiotemporal Expression Profiles of AmVgR

**Figure 3:**
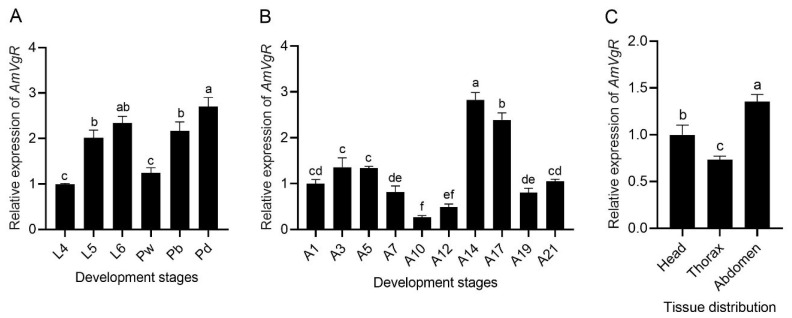
Relative expression levels of *AmVgR* at different developmental and growth stages as well as in different tissues. (**A**) Expression of *AmVgR* in four-day-old to six-day-old larvae (L4–L6), white-eye pupae (Pw), brown-eye pupae (Pb), black-eye pupae (Pd) of worker bees. (**B**) Expression of *AmVgR* in one-day-old to twenty-one-day-old adults (A1–A21) of worker bees. (**C**) Expression of *AmVgR* in the head, thorax, and abdomen of worker bees. The data are presented as the mean ± SEM. Distinct letters in the figure denote statistically significant differences (*p* < 0.05) as determined by Duncan’s multiple-range test, while identical letters indicate no significant difference.

To assess the expression patterns of *AmVgR* in *Apis mellifera*, we explored its expression levels at different developmental stages and in various tissue types. During the larval and pupal stages, the expression level of *AmVgR* exhibited a gradual upward trend (Figure 3A). After the emergence of adult bees, the expression levels of *AmVgR* first increased and then decreased. The peak expression was detected at 14 days after emergence (Figure 3B). This particular age coincides with the foraging age of bees [43]. Additionally, we evaluated the expression levels of *AmVgR* in diverse tissues of foraging bees. Our findings revealed that the expression of *AmVgR* was notably higher in the abdominal tissues than in the head and thoracic tissues (*p* < 0.05). These results suggest that *AmVgR* might be essential for growth, development, and behavioral changes in *Apis mellifera*.

### 3.4. AmVgR Gene Expression Profile Under Different Stress Conditions

**Figure 4:**
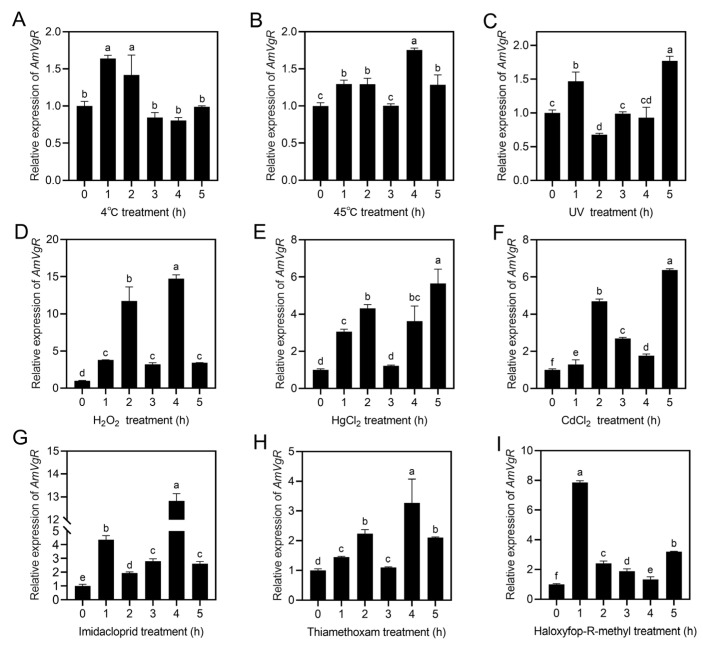
Expression characteristics of *AmVgR* under different stresses. Treatments included (**A**) 4 °C, (**B**) 45 °C, (**C**) UV irradiation, (**D**) H_2_O_2_, (**E**) HgCl_2_, (**F**) CdCl_2_, (**G**) imidacloprid, (**H**) thiamethoxam, and (**I**) haloxyfop-R-methyl. The data are presented as the mean ± SEM. Distinct letters in the figure denote statistically significant differences (*p* < 0.05) as determined by Duncan’s multiple-range test, while identical letters indicate no significant difference.

To determine the response of *AmVgR* to abiotic stressors in *Apis mellifera*, we subjected the bees to a range of stress treatments, which included extreme temperatures (45 °C and 4 °C), ultraviolet radiation, heavy metals (CdCl_2_ and HgCl_2_), and pesticides (imidacloprid, thiamethoxam, and haloxyfop-R-methyl). We assessed the expression levels of *AmVgR* under these stress conditions using RT-qPCR. The results showed that *AmVgR* expression was upregulated in response to both high and low temperatures (*p* < 0.05) (Figure 4A,B). After UV irradiation, *AmVgR* expression initially increased significantly, before decreasing (*p* < 0.05) (Figure 4C). Following treatments with H_2_O_2_, HgCl_2_, and CdCl_2_, *AmVgR* expression was also significantly upregulated (*p* < 0.05) (Figure 4D–F). In addition, exposure to the three different pesticides imidacloprid, thiamethoxam, and haloxyfop-R-methyl led to significant upregulation of *AmVgR* expression, with similar expression patterns observed (*p* < 0.05) (Figure 4G–I). These findings suggest that the *AmVgR* gene in *Apis mellifera* exhibits responsiveness to a range of abiotic stressors and may play a critical role in bees’ defense mechanisms against oxidative stress induced by adverse environmental conditions.

### 3.5. AmVgR Protein Expression Under Abiotic Stress Conditions

**Figure 5:**
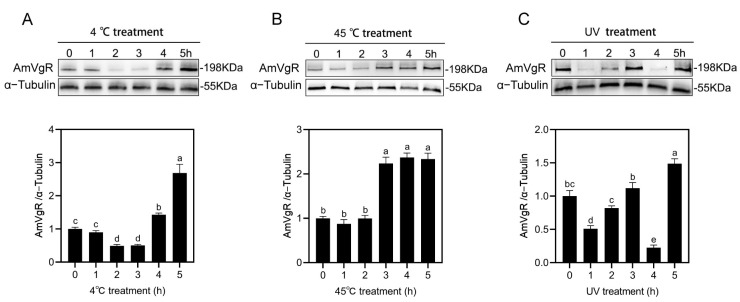
Analysis of AmVgR protein expression under different abiotic stresses. (**A**) Low-temperature treatment at 4 °C. (**B**) High-temperature treatment at 45 °C. (**C**) UV irradiation treatment. The data are presented as the mean ± SEM. Distinct letters in the figure denote statistically significant differences (*p* < 0.05) as determined by Duncan’s multiple-range test, while identical letters indicate no significant difference.

We validated the effects of high temperature (45 °C), extreme cold (4 °C), and UV radiation stress on *AmVgR* in *Apis mellifera* at the protein level. The results of the WB experiment showed that after the low-temperature stress treatment (4 °C), the expression of the *AmVgR* protein in honeybees first decreased and then increased. It reached the lowest level 2–3 h after the treatment and then gradually increased, reaching the highest expression level at 5 h, which was 2.5 times the initial value (Figure 5A). After the high-temperature stress treatment (45 °C), the expression of the AmVgR protein remained stable in the first 1–2 h, but increased rapidly after 3–5 h, reaching approximately 2.1 times that at 0 h. The results showed that following cold stress (4 °C) treatment, AmVgR protein expression in bees initially decreased and then increased, reaching a minimum 2–3 h after treatment and gradually rising thereafter, with the highest expression observed at 5 h, which was 2.5 times the initial value (Figure 5A). After high-temperature stress (45 °C) treatment, AmVgR protein expression remained stable during the first 1–2 h but rapidly increased after 3–5 h, reaching approximately 2.1 times the level at 0 h (Figure 5B). When bees were exposed to UV irradiation for 1 h, AmVgR protein expression significantly decreased, followed by gradual recovery after 2–3 h, with the lowest expression observed at 4 h, and the highest level at 5 h post-treatment (Figure 5C). These findings demonstrate that AmVgR protein responds to abiotic stress, further confirming its role in the oxidative stress response in *Apis mellifera*.

### 3.6. Study of the Effects of Knocking Down AmVgR on the Antioxidant Capacity in Apis mellifera

**Figure 6:**
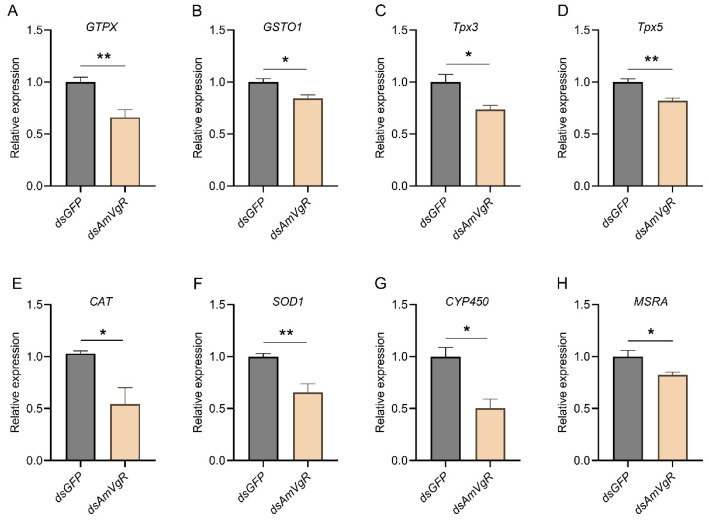
Antioxidant-related gene expression after *AmVgR* silencing, including (**A**) *GTPX*, (**B**) *GSTO1*, (**C**) *Tpx3*, (**D**) *Tpx5*, (**E**) *CAT*, (**F**) *SOD1*, (**G**) *CYP450*, (**H**) *MSRA*. * *p* < 0.05 and ** *p* < 0.01.

**Figure 7:**
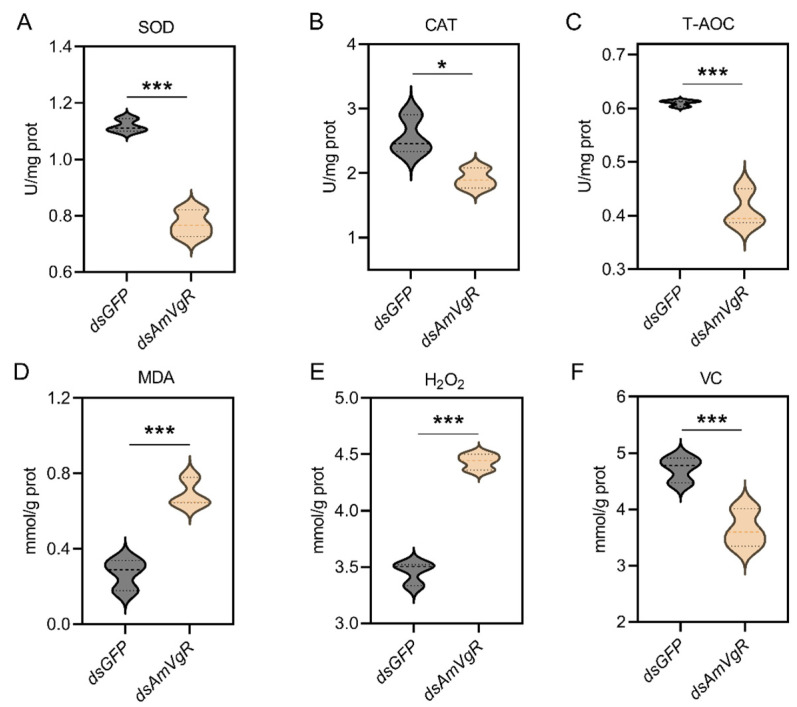
Antioxidant enzyme activity and antioxidant substance content after *AmVgR* silencing. (**A**) SOD, (**B**) CAT, (**C**) T-AOC, (**D**) MDA, (**E**) H_2_O_2_, and (**F**) VC. * *p* < 0.05 and *** *p* < 0.001.

To further verify the function of *AmVgR* in the stress response of *Apis mellifera*, we silenced the gene by feeding the corresponding dsRNA to A16 forager bees. The results demonstrated that feeding *dsAmVgR* significantly reduced the transcriptional level of the *AmVgR* gene (*p* < 0.05) (Figure S3). We further analyzed the expression levels of antioxidant genes, antioxidant enzyme activities, and the accumulation of oxidative substances in bees following *AmVgR* gene silencing. After feeding *dsAmVgR*, the expression levels of antioxidant genes, including *GTPX*, *GSTO1*, *Tpx3*, *Tpx5*, *CAT*, *SOD1*, *CYP450*, and *MSRA*, were significantly downregulated (*p* < 0.05) (Figure 6). Additionally, the activities of the SOD and CAT enzymes were markedly reduced (*p* < 0.05) (Figure 7A,B), and the total antioxidant capacity (T-AOC) was also significantly decreased (*p* < 0.05) (Figure 7C). Concurrently, the levels of oxidative substances, such as H_2_O_2_ and MDA, were significantly elevated (*p* < 0.05) (Figure 7D,E), while the content of the antioxidant vitamin C (VC) was notably reduced (*p* < 0.05) (Figure 7F). These findings indicate that silencing the *AmVgR* gene led to the downregulation of multiple antioxidant genes, reduced antioxidant enzyme activities, and diminished the total antioxidant capacity, which was accompanied by increased accumulation of oxidative substances. This suggests that *AmVgR* plays a critical role in the oxidative stress defense mechanism in *Apis mellifera*.

### 3.7. The Knockdown of AmVgR Reduces Antioxidant Stress Resistance in Apis mellifera

**Figure 8:**
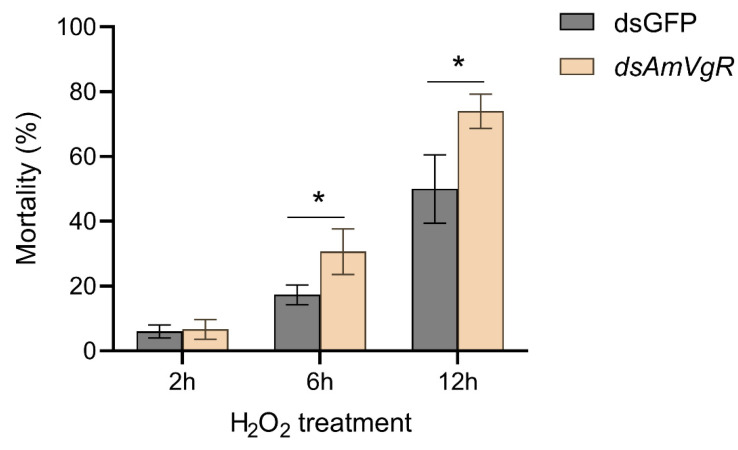
Oxidative stress resistance in *Apis mellifera* after silencing *AmVgR*. * *p* < 0.05.

To explore the functional significance of *AmVgR* in the antioxidant stress responses of *Apis mellifera*, we subjected bees with silenced *AmVgR* expression to H_2_O_2_-induced oxidative stress. The results indicated that the bees treated with *dsAmVgR* exhibited significantly elevated mortality rates when compared with the *dsGFP* control group. Specifically, after six hours of exposure to H_2_O_2_, the mortality rate in the *dsAmVgR* group was 50.00 ± 10.58%, whereas the control cohort demonstrated a considerably lower rate of 13.77 ± 3.06%. After twelve hours of treatment, the mortality rate in the *dsAmVgR* group further increased to 74.00 ± 5.29%, compared to 30.67 ± 7.02% in the control group (Figure 8). The substantial rise in mortality associated with the silencing of the *AmVgR* gene during H_2_O_2_-induced oxidative stress highlights the critical function of *AmVgR* within the antioxidant defense mechanisms of *Apis mellifera*.

## 4. Discussion

The *VgR* protein belongs to LDLR family and regulates the entry of vitellogenin into oocytes through endocytosis. Members of the *VgR* family are found in all oviparous vertebrates and invertebrates [23,44]. In our study, we cloned the full-length cDNA of the *AmVgR* gene and analyzed the molecular characteristics of the protein. A phylogenetic analysis indicated that *AmVgR* clusters within the Hymenoptera branch and shares homology with other *VgR* proteins in Hymenoptera, showing the highest homology with the VgR of *Apis cerana cerana* (Figure 1A). A protein structural analysis revealed that *AmVgR* shares several typical domains with *VgR* proteins of other insects, such as the LDLR domain, EGF-like domain, calcium-binding EGF-like domain, and low-density lipoprotein receptor YWTD domain (Figure 1B,C). Similar to most insect *VgRs*, *AmVgR* contains four cysteine-rich LDLRA repeats in the first binding site and eight in the second binding site (Figure 1C). This arrangement differs from the *VgR* structures in other insects, such as the silkworm, cotton bollworm, and cutworm, which have four and seven repeats, and the psocid and American cockroach, which have five and eight repeats [45]. Additionally, *AmVgR* has an 18-amino acid signal peptide at its N-terminus, MSRNLLVFFVLTNFYCSS, which is not present in all insect *VgRs*, such as those of the Mediterranean fruit fly and the oriental fruit fly (Figure S2) [46]. These findings indicate that while *VgRs* in insects are highly conserved and homologous, the number and arrangement of their domains exhibit species-specific characteristics.

Analysis of the *AmVgR* promoter sequence indicated the existence of cis-acting elements related to environmental stress and immune responses (Figure 2), including the TATA box, CREB, AP1, HSF, Sox-5, and Oct-1 [47,48]. The TATA box is a fundamental component of the promoter region that determines the transcription start site. Genes containing a TATA box are predominantly linked to environmental stress and exhibit corresponding responses [49]. *CREB* is a critical transcription factor that interacts with multiple intracellular signaling pathways and regulates transcriptional activity through phosphorylation [50]. *HSF* modulates the expression of genes such as *hsp104*, *hsp90*, and *hsp70* under stress conditions [38]. Research indicates that enhanced *HSF-1* activity can mitigate mitochondrial damage, inhibit protein toxicity, and extend the lifespan [39]. *Oct-1* and *Sox-5* play crucial roles in cell fate determination, tissue formation, and organ development [41,42]. These findings suggest that *AmVgR* may play important roles in the growth and development of bees, as well as in their antioxidant stress responses.

The expression of *VgR* genes is thought to be highly tissue-specific and only present in the ovaries of female insects [23,51]. However, the *VgR* gene in the parasitoid wasp *Pteromalus puparum* is expressed not only in females but also in males, whereas the *VgR* gene is only expressed during ovarian development in other insects such as the multicolored Asian lady beetle (*Harmonia axyridis*), the migratory locust (*Locusta migratoria*), and the oriental fruit fly (*Bactrocera dorsalis*) [45,52,53,54]. In *Apis mellifera*, *AmVgR* expression has been detected in the hypopharyngeal glands, ovaries, fat body, and other tissues [29]. In our study, *AmVgR* gene expression was observed across various developmental stages and all tested tissues of *Apis mellifera*. Notably, the gene exhibited significantly higher expression in bees at 14 and 17 days of age, which corresponds to the peak foraging period (Figure 3B). Additionally, the expression levels were notably higher in the abdomen compared to the head and thorax (Figure 3C), suggesting a potential relationship with the pleiotropic biological functions of its ligand [29,55].

Honeybees play a crucial role in agricultural productivity and in maintaining the natural ecological balance [56,57]. However, their foraging activities often expose them to abiotic stresses such as extreme weather, insecticides, and heavy metals. Previous studies have shown that high temperatures can lead to water loss, ionic changes within cells, and oxidative stress, ultimately resulting in mortality [58]. Pollutants such as insecticides and heavy metals can be transferred through bees’ foraging behavior and accumulate in the colony, negatively impacting honeybee health [59,60,61,62]. In our study, exposure to high and low temperatures, UV radiation, pesticides, and heavy metals resulted in varying degrees of increased or decreased expression of both the *AmVgR* gene and the protein in *Apis mellifera* (Figure 4 and Figure 5). These findings suggest that *AmVgR* may play a significant role in *Apis mellifera*’s response to environmental stressors [7]. Research has shown that the expression of *VgR* is also influenced by environmental factors. For example, variations in temperature and exposure to pesticides can influence the *VgR* expression levels, consequently impacting the physiological conditions and reproductive capacity of insects [63,64]. Furthermore, *VgR* is associated with the antioxidant capacity of insects, as it enhances the activity of antioxidant enzymes and reduces the intracellular ROS levels. This process mitigates the cellular damage caused by oxidative stress and protects cells from apoptosis [65]. In species such as the Oriental armyworm (*Mythimna separata*) and the melon fly (*Zeugodacus cucurbitae*), environmental stresses like ultraviolet radiation or brief periods of elevated temperature can trigger the upregulation of *VgR* gene expression [66,67]. This upregulation may represent an adaptive response, whereby *VgR* expression is increased to bolster the insect’s defense mechanisms against environmental stressors.

In this study, we observed that silencing the *AmVgR* gene in honeybees led to a significant downregulation in the expression of antioxidant and detoxification genes, including *GSTO1*, *SOD1*, and *CYP450* (Figure 6). *GSTO1* is implicated in intracellular detoxification and antioxidant processes, facilitating the catalytic conjugation of glutathione with various electrophilic compounds, thereby enhancing the metabolism and excretion of these harmful substances [68]. *SOD1* plays a crucial role in scavenging intracellular ROS by catalyzing the dismutation of superoxide anions into hydrogen peroxide and oxygen [69]. The *CYP450* enzyme family is involved in the metabolism and detoxification of a broad spectrum of endogenous and exogenous compounds [70]. The observed downregulation of these genes suggests that *AmVgR* may modulate their expression at the transcriptional level. Consequently, the loss of *AmVgR* function impairs the normal expression of these genes, leading to a diminished cellular capacity for antioxidant defense and detoxification.

Meanwhile, we observed a significant decrease in the activities of antioxidant enzymes such as CAT and SOD (Figure 7A–C). CAT facilitates the decomposition of hydrogen peroxide into water and oxygen and operates synergistically with SOD to maintain the intracellular ROS equilibrium [71]. The diminished activity of these antioxidant enzymes may further aggravate the accumulation of intracellular ROS, resulting in an elevated presence of oxidative damage markers such as H_2_O_2_ and MDA (Figure 7D,E) [72,73]. MDA, a byproduct of lipid peroxidation, serves as an indicator of oxidative damage to the cell membrane [74,75]. Additionally, the concentration of the antioxidant compound VC decreased MDA (Figure 7F), potentially due to heightened intracellular oxidative stress, wherein VC is rapidly consumed to counteract ROS, and cellular mechanisms fail to replenish it promptly [74]. These findings suggest that the silencing of *AmVgR* may disturb the redox homeostasis in honeybee cells, precipitating a state of oxidative stress. After inducing oxidative stress with H_2_O_2_, the survival rate of the honeybees decreased significantly (*p* < 0.05) (Figure 8), which further confirmed the indispensable role of the *VgR* gene in the oxidative stress response of honeybees. H_2_O_2_ is a common ROS that can penetrate the cell membrane and generate more reactive hydroxyl radicals inside the cell, causing damage to intracellular biomacromolecules such as DNA, proteins, and lipids [76,77]. Therefore, when the *AmVgR* gene is silenced, the antioxidant system of honeybees is damaged and unable to effectively scavenge ROS such as H_2_O_2_, which leads to intensified cell damage and ultimately affecting the survival ability of honeybees. This shows that the *VgR* gene is crucial for maintaining the normal function of the antioxidant defense system of honeybees. It may help honeybees cope with oxidative stress and protect cells from oxidative damage by regulating the expression of antioxidant and detoxification genes and the activities of antioxidant enzymes.

## 5. Conclusions

In summary, *AmVgR* plays a critical role in oxidative stress responses in *Apis mellifera* exposed to abiotic stresses such as high temperatures, pesticides, and heavy metals. Our study provides preliminary insights into the sequence, phylogeny, and expression patterns of *AmVgR* in *Apis mellifera*. Additionally, silencing *AmVgR* significantly reduced the total antioxidative capacity of the bees and increased their mortality rates under oxidative stress. These findings underscore the pivotal role of *AmVgR* in *Apis mellifera* adaptation to environmental pressures. However, further research is needed to elucidate the mechanisms by which *AmVgR* mediates antioxidative capacity and other physiological functions in *Apis mellifera*.

## 6. Limitations and Future Perspectives

Although the study found that the silencing of the *AmVgR* gene affected the expression of antioxidant genes and the activity of antioxidant enzymes, the specific molecular pathways through which *AmVgR* regulates these genes and enzymes remain unclear. For example, it is unknown whether *AmVgR* directly binds to the promoter regions of antioxidant and detoxification genes to regulate their transcription or indirectly affects gene expression and enzyme activity through other signaling pathways.

Future investigations into *AmVgR* should prioritize a comprehensive analysis of its diverse functions and mechanisms within insect physiology and ecology. While current studies have highlighted the significant role of *VgR* in insect development and reproduction [78], the precise molecular mechanisms and signaling pathways remain inadequately understood and warrant further investigation. Additionally, research into the application of *VgR* as a biomarker and target for insect control encounters several challenges, such as technological limitations, the selection of appropriate research models, and the assessment of ecological impacts. Advancing research in this area necessitates interdisciplinary collaboration, incorporating insights from molecular biology, ecology, and agricultural science to foster the comprehensive development of *VgR* applications. Concurrently, researchers must also consider the potential role of *VgR* in addressing global climate change and ecosystem services, thereby contributing to broader ecological protection and sustainable development objectives.

## Data Availability

The raw data supporting the conclusions of this article will be made available by the authors on request.

## Supplementary Materials

The following supporting information can be downloaded at: https://www.mdpi.com/article/10.3390/insects16070650/s1. Figure S1: *AmVgR* gene cloning and expression of *AmVgR* in *E. coli*; Figure S2: signal peptide prediction for the sequence of *AmVgR*; Figure S3: detection of the silencing effect of *AmVgR* in worker bees; Table S1: pesticide and heavy metal information; Table S2: Information on the primers used in this study; Table S3: PCR amplification conditions; Table S4: Quantitative real-time PCR procedure; Table S5: Genetic information on phylogenetic tree species.
