# Supplementary material for: Functional Elucidation of Vitellogenin receptor Activity in Apis mellifera in Response to Abiotic Stress
Source: Insects. 2025 Jun 21;16(7):650. doi: 10.3390/insects16070650 (PMC12295193; doi:10.3390/insects16070650)
Supplement: Supplementary file 1 [file insects-16-00650-s001.zip › insects-3638474-supplementary.pdf]

Table S1

Pesticide and heavy metal information.

Reagents	Fineness	Manufacturers
H_2O_2	30%	Laiyang City Kant language industry Co., LTD, China
$HgCl_2$	99.5%	Tongren Chemical Reagent Co. Ltd., Guizhou, China
$CdCl_2$	99%	Kaitong Chemical Reagent Co. Ltd., Tianjin, China
Imidacloprid ($C_9H_{10}ClN_5O_2$, CAS: 138261-41-3;105827-78-9)	70%	Guanlong Agrochemical Co. Ltd., Hebei China
Thiamethoxam ($C_8H_{10}ClN_5O_3S$, CAS: 153719-23-4)	70%	Guihe Bioscience Co. Ltd., Shandong, China
Haloxyfop-P-methyl ($C_{16}H_{13}ClF_3NO_4$, CAS: 721619-32-0)	108 g/L	Kexin Bioscience Co. Ltd., Shandong China

Table S2

Information on the primers used in this experiment.

Purpose	Gene name	Primer Sequences (5′-3′)
Gene cloning	*VgR1-F*	ATGTCTCGCAATTTACTCGTGTTC
	VgR1-R	TCACTCTCATCGGAACCATCTG
	VgR2-F	GACTGCACCGATCTTTCTGA
	VgR2-R	GCTGTTGCATTATTGCCCAT
	VgR3-F	TCCAGCAGTATTGAGGTATGC
	VgR3-R	GCTATATACAGTTAGATTTTGGTTC
RT-qPCR	*β-actin-F*	TTATATGCCAACACTGTCCTTT
	β-actin-R	AGAATTGATCCACCAATCCA
	AmVgR-F	TGTCACGAGGGAGCTTGCATA
	AmVgR-R	ACAAGTCCCGATTTCACACGC
	SOD1-F	AAACTATTCAACTTCAAGGACC
	SOD1-R	CACAAGCAAGACGAGCACC
	CAT-F	GTCTTGGCCCGAACAATTTG
	CAT-R	CATTCTCTAGGCCCACCAAA
	GSTO1-F	CATTCTTTCATGGTAATTCTCCTGGC
	GSTO1-R	TTAATCAGTAATCAAATCATATTGTGG
	Tpx3-F	CCTGCACCTGAATTTTCCGG
	Tpx3-R	CCTGCACCTGAATTTTCCGG
	Tpx5-F	GGGGTATTCTATTTTCGCATCCA
	Tpx5-R	CCATTTACGATGAGAATCGACTGA
	GTPX-F	CGACAACTATAAGGAAGCGAAA

	GTPX-R	AGATAGAAAAACGTCTTCGCCT
	CYP450-F	AATGGGATTCCGTACAGCAA
	CYP450-R	GTAGTTGCATTTTCGGCGAAACCTC
	MSRA-F	CAAAATGCCAGGACAATTCG
	MSRA-R	CCAGAAACATCCCATTCCAA
RNAi	*dsAmVgR-F*	TAATACGACTCACTATAGGGCTGCACCGATCTTTCTGACG
	dsAmVgR-R	TAATACGACTCACTATAGGGTTCATCCGATCCATCGCCAC
	dsGFP-F	TAATACGACTCACTATAGGGCGAAGTGGAGAGGGTGAAGGTGA
	dsGFP-R	TAATACGACTCACTATAGGGCGAGGTAAAAGGACAGGGCCATC

Table S3

PCR amplification conditions.

Primer pair	Amplification conditions
Am*VgR-F*/Am*VgR-R*	10 min at 95°C, 40 s at 95°C, 40 s at 47°C, 55 s at 72°C for 35 cycles, 10 min at 72°C

Table S4

Quantitative real-time PCR procedure.

Step	Number of Cycles	Temperature	Time
Step1	1 cycle	95°C	30 s
Step2	40 cycles	95°C	5 s
		60°C	30 s
Step3	Dissociation stage		

Table S5

Genetic information of phylogenetic tree species.

Species name	Gene number	Species name	Gene number
Apis mellifera	XP_026295652.1	*Bombus lantschouensis*	QGZ98018.1
Apis cerana cerana	PBC31775.1	*Megachile rotundata*	XM_003704612
Apis dorsata	XP_006610571.1	*Solenopsis invicta*	NM_001304596.1
Apis laboriosa	XP_043793917.1	*Bemisia tabaci*	HM017828.2
Apis florea	XP_012350206.1	*Nilaparvata lugens*	GU72397
Colaphellus bowringi	MH104867.1	*Aedes aegypti*	L77800
Diabrotica virgifera	AQS83398.1	*Tuta absoluta*	MZ682118
Periplaneta americana	AB077047.2	*Bombyx mori*	ADK94452
Locusta migratoria	QCX5737.1	*Helicoverpa armigera*	KC181922.2
Bactrocera dorsalis	JX469118	*Spodoptera exigua*	KT899978.1
Drosophila melanogaster	U13637	*Spodoptera litura*	GU983858.1
Penaeusmonodon	ABW79798.1	*Macrobrachium rosenbergii*	ADK55596.1
Pandalusjaponicus	AHL26192.1	*Bombus terrestris*	XP_003402703.1

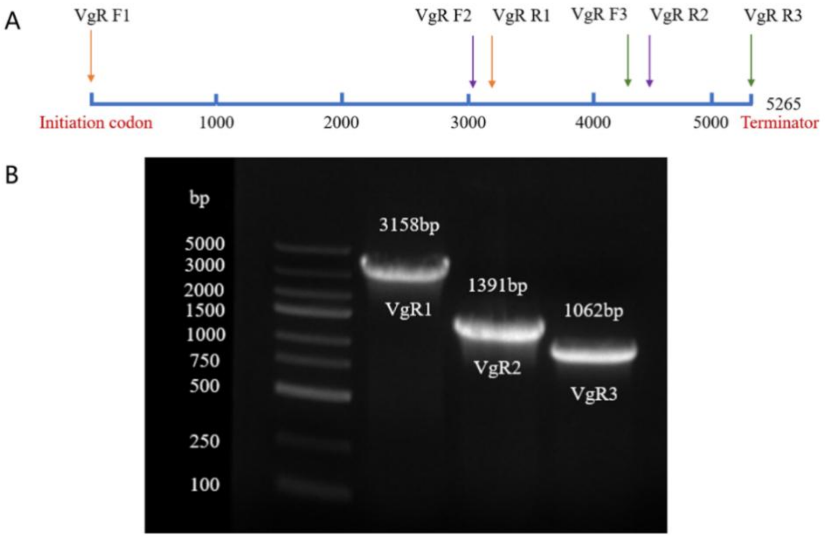

Figure S1. *AmVgR* gene cloning and expression of *AmVgR* in E. coli. (A) Schematic representation of POR amplification of the *AmVgR*. (B) PCR amplification electropherogram of *AmVgR*.

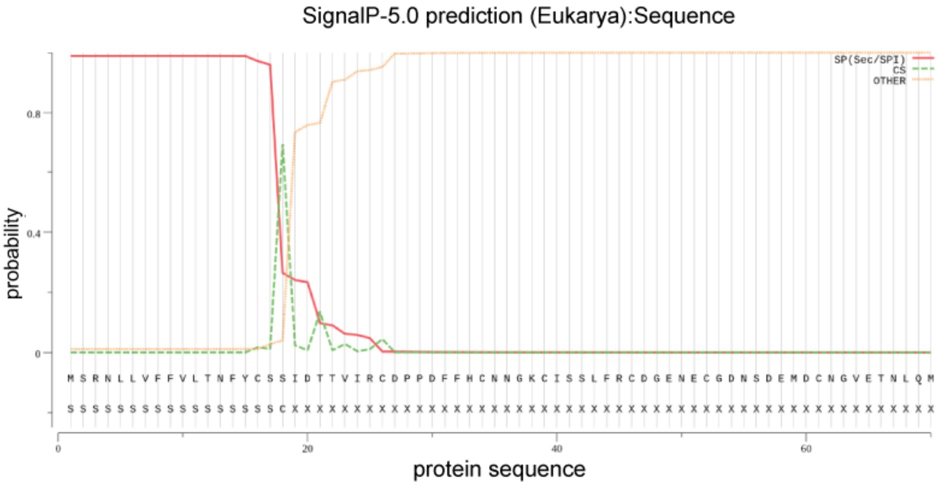

Figure S2. Signal peptide prediction of the sequence of *AmVgR*.

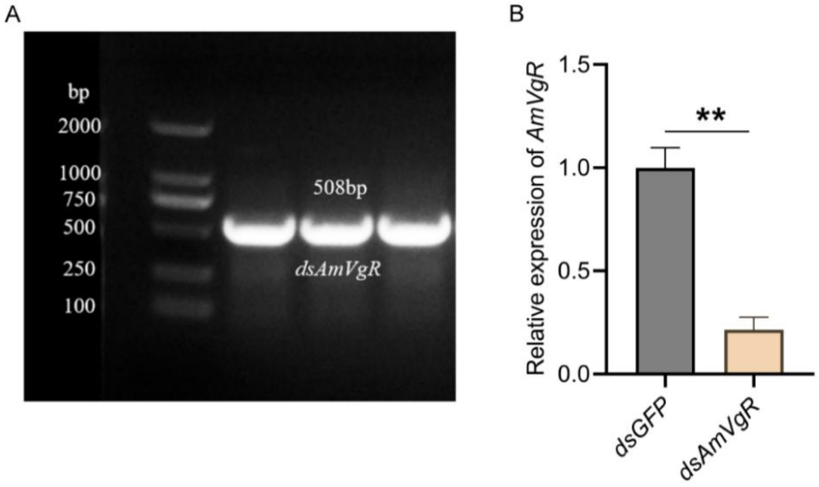

Figure S3. Detection of silencing effect of *AmVgR* in worker bees. (A) Gel electrophoresis image of the *dsAmVgR* double strand. (B) Expression levels of the *AmVgR* gene after silencing. **P < 0.01.
